# The application of iliac fascia space block combined with esketamine intravenous general anesthesia in PFNA surgery of the elderly: A prospective, single-center, controlled trial

**DOI:** 10.1515/med-2023-0783

**Published:** 2023-08-31

**Authors:** Xuandong Jia, Xingzhi Liao, Maitao Zhou

**Affiliations:** Department of Anesthesiology, The 904th Hospital of the Joint Logistic Support Force of PLA, Wuxi 214000, Jiangsu Province, China

**Keywords:** proximal femoral nail antirotation, retain spontaneous breathing, iliac fascia space block, esketamine

## Abstract

To observe the effect of iliac fascia space block combined with esketamine intravenous general anesthesia in proximal femoral nail antirotation (PFNA) of the elderly. Eighty elderly patients who underwent PFNA were randomly divided into experimental group and control group. In the experimental group, iliac fascial block combined with esketamine and propofol intravenous general anesthesia was used to keep spontaneous breathing. The control group used iliac fascia block combined with remifentanil and propofol intravenous general anesthesia to maintain spontaneous breathing. Record important indexes such as heart rate (HR), mean arterial pressure (MAP), pulse oxygen saturation (SpO_2_), visual analogue score (VAS) scores, etc. at different moment during the operation. Trial data showed that there were significant differences in HR, MAP, and SpO_2_ between the two groups at the beginning of operation, and there was no significant difference in VAS scores between the two groups at each moment after surgery, and there were significant differences in the number of vasopressor applications, length of hospital stay, and QoR-15 scores between the two groups, and there were significant differences in the incidence of total adverse reactions and the incidence of hypotension. The trial indicated that patients in the experimental group have more stable hemodynamics and lower stress response, which is conducive to rapid recovery after surgery.

## 1 Introduction

Intertrochanteric fractures of the femur occur more often in the elderly and are often treated with proximal femoral nail antirotation (PFNA) internal fixation [1,2]. At present, this procedure mostly uses neuraxial anesthesia or general anesthesia. Previous studies have shown that the above two anesthesia methods have good anesthesia effects on elderly patients with intertrochanteric fractures, and there are no obvious differences [3], but neuraxial anesthesia has problems such as difficult control of anesthesia plane, difficult operation, and easy to cause secondary injury in position [4]. General anesthesia alone interferes greatly with the patient’s physiology and easily increases the risk of postoperative complications [5]. The choice of the best anesthesia modality remains controversial, and safe and reliable anesthesia methods remain one of the key factors in the success of PFNA, as well as the use of new drugs, new anesthesia techniques, or combinations. Therefore, this article studies the application of fascia iliac block combined with esketamine intravenous general anesthesia in elderly patients with PFNA, so as to provide a reference for further improving the management of perioperative anesthesia.

## 2 Material and methods

### 2.1 General information

A total of 80 elderly patients who underwent elective PFNA internal fixation from November 2021 to August 2022 were randomly divided into experimental group (S group) and control group (C group), with 40 cases in each group. In the experimental group, fascial iliac block was used combined with esketamine and propofol intravenous general anesthesia to preserve the patient’s spontaneous breathing. The control group used fascial iliac block combined with refentanil, propofol, and laryngeal mask to assist spontaneous breathing. Inclusion criteria: elective surgery patients who can actively cooperate, have normal heart, liver, kidney and lung function before surgery, and are not allergic to the drugs used in this study. Age > 65 years old, regardless of gender, body mass index (BMI) 18–28 kg/m^2^, American society of anesthesiologists (ASA) score II–III. Exclusion criteria: patients with neurological, psychiatric diseases, hematologic, immune system, respiratory system and other major diseases, history of allergy or addiction to esketamine and propofol, and contraindications to surgery. Approved by the ethics committee of our hospital, the patient signed the informed consent form before surgery.

### 2.2 Anesthesia method

Both groups routinely fasted from eating and drinking. All patients were given intravenous access, nasal cannula for 2 L/min of oxygen, and routine monitoring of blood pressure (BP), heart rate (HR), and pulse oxygen saturation (SpO_2_). Under the guidance of ultrasound, the iliac fascia space block (modified high iliac fascia space block-subvascular method) was performed, and the specific process could be described as follows. First, placed the sterile-sheathed ultrasound probe on the position, which was on the line connecting the navel and the anterior superior iliac spine on the side of the anterior superior iliac spine. Once the iliacus muscle and anterior superior iliac spine were identified, the outer end of the probe was adjusted to approximately 15° inward rotation. Then, using the in-plane technique, the tip of the ultrasonic probe was placed under the deep circumflex iliac artery and 30 mL of 0.375% ropivacaine hydrochloride and 0.5 µg/kg dexmedetomidine were injected into that area. After injection, the deep circumflex iliac artery moved upwards and the iliacus muscle moved downwards, followed by invasive arterial monitoring and Bis monitoring.

After the successful block, the patients in S group were maintained with the dose of 0.3 mg/kg/h of esketamine and the dose of 2–4 mg/kg/h of propofol under micropump anesthesia, and 0.3–0.5 mg/kg of esketamine was slowly injected intravenously at the beginning of surgery (before skin incision). During the operation, if the patient had a body movement reaction, the anesthesiologist could add 0.25 mg/kg of esketamine in a single dose according to the patient’s condition. The C group pumped refentanil and propofol, in target-controlled infusion mode, the Minto model and Marsh model were selected, respectively, and 2.5–4.5 ng/mL of refentanil and 2–3 μg/mL of propofol were loaded first, the eyelash reflex of the patient to be observed disappeared, and when the Bis value of the anesthesia depth was below 60, the target concentration of refentanil and 1–2 μg/mL propofol was maintained, and a laryngeal mask was inserted, and spontaneous breathing was retained (spontaneous breathing mode of the anesthesia machine). After receiving a load dose of remifentanil and propofol, patients in C group might experience temporary respiratory depression as the blood concentration increased. At this moment, it was necessary to change to controlled breathing mode and observe the waveform of end-tidal carbon dioxide. After the patient had spontaneous breathing, the anesthesia machine was adjusted to spontaneous breathing mode, and the Bis value was maintained at 40–60 during the operation. When the patient’s BP was below 20% of the preoperative basal value, the vasoactive drug ephedrine or metahydroxylamine was given. After the operation, if the visual analogue score (VAS) of patients in both groups was greater than 5, both groups were given 15–30 mg of ketorolone for pain relief.

### 2.3 Observation index

HR, mean arterial pressure (MAP), and SpO_2_ at the time of admission (*T*0), at the beginning of surgery (*T*1), 15 min from the beginning of surgery (*T*2), and at the end of surgery (*T*3); VAS scores of 2 h (*T*4), 6 h (*T*5), 12 h (*T*6), 24 h (*T*7), and 48 h (*T*8) were recorded postoperatively. The number of intraoperative vasopressor doses, the quality of recovery at 24 h after surgery, and the length of hospital stay were recorded. Assessment of postoperative 24 h recovery quality (QoR-15) score (15 quality of recovery questionnaire 15 (QoR-15), a total score of 150 points, each score is scored by 0–10 points, the sum is taken as the final evaluation result, the higher the total score, the better the patient’s recovery quality [6]). Adverse effects such as nausea and vomiting, hypotension, hyperalgesia, and delirium were recorded.

### 2.4 Statistical analysis

The statistical data are analyzed using SPSS 22.0. The measurement data were expressed by ( \[\bar{x}\pm s]\] ), and the *t-*test was used for comparison. The counting data are represented by cases or percent (%), and the *χ* ^2^ test was used for comparison. *P* < 0.05 was statistically significant.

## 3 Results

There were no significant differences in age, BMI, and ASA scores between the two groups (*P* > 0.05) (Table 1).

**Table 1:** Comparison of the general condition of the two groups

General information		S	C	*t* (*χ* ^2^)	*P*
Age (years)		75.86 ± 7.60	76.75 ± 7.70	−0.52	0.605
BMI (kg/m^2^)		22.51 ± 1.84	21.82 ± 2.13	1.559	0.123
ASA	Class II	8	10	0.287	0.592
	Class III	32	30		

**Figure 1:**
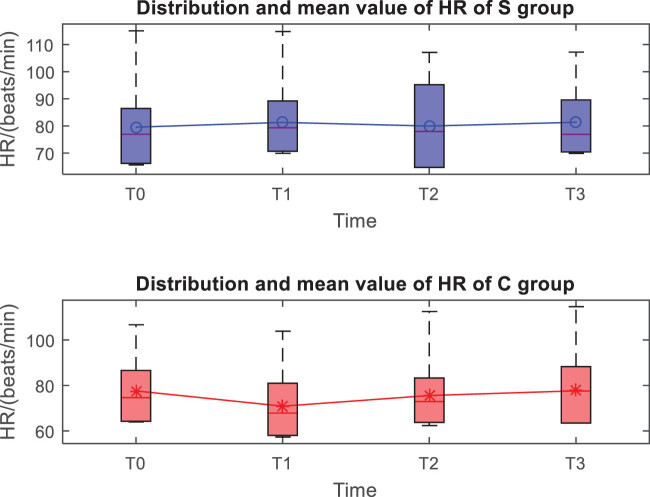
Distribution and mean value of HR of the two groups.

**Figure 2:**
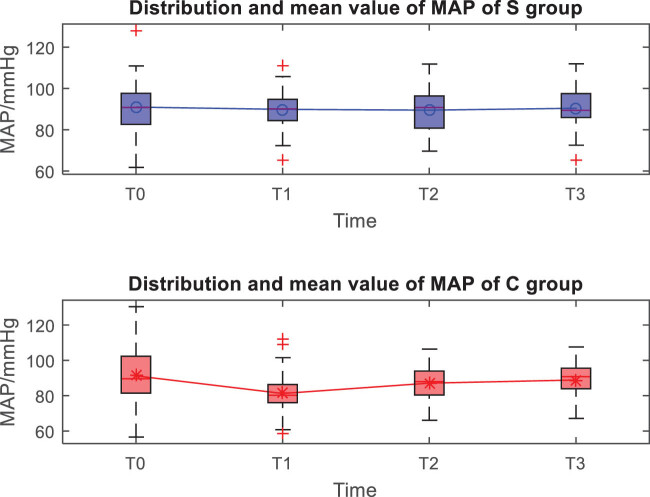
Distribution and mean value of MAP of the two groups.

**Figure 3:**
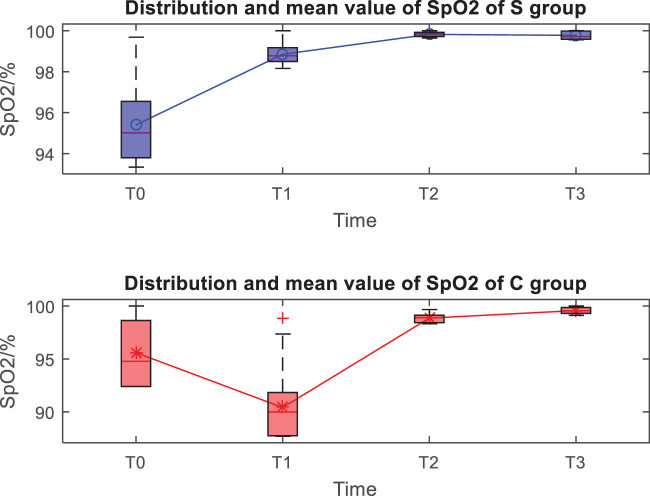
Distribution and mean value of SpO_2_ of the two groups.

The distribution and mean value of HR, MAP, and SpO_2_ of the two groups at *T*0, *T*1, *T*2, and *T*3 are shown in Figures 1–3. Taking the distribution of the measured values of HR of the S group at *T*0 as an example, the box diagram contains the upper adjacent, lower adjacent, and median of the distribution of the measured values. The box part represents the interval of most measured values.

At the *T*1 time, the differences in HR, MAP, and SpO_2_ between the two groups are statistically significant (*P* < 0.05), while the differences between the two groups at other times are not statistically significant (*P* > 0.05) (Table 2).

**Table 2:** Comparison of HR, MAP, and SpO_2_ at different time points between the two groups ( \[\bar{x}\pm s]\] )

Index	Group	Time			
		*T*0	*T*1	*T*2	*T*3
HR (beats/minute)	S (*n* = 40)	79.44 ± 13.78	81.33 ± 11.43^b^	79.99 ± 15.28	81.17 ± 11.28
	C (*n* = 40)	77.45 ± 13.48^a^	70.97 ± 13.68^a,b^	75.43 ± 13.07	77.52 ± 14.06
*t*		0.655	4.416	1.435	1.280
*P*		0.514	0.001	0.155	0.204
MAP (mmHg)	S (*n* = 40)	90.82 ± 12.37	90.06 ± 9.79^b^	89.63 ± 10.27	90.37 ± 10.39
	C (*n* = 40)	91.12 ± 15.80^a^	81.18 ± 11.93^a,b^	87.01 ± 10.16	88.68 ± 9.62
*t*		−0.096	3.904	1.146	0.755
*P*		0.924	0.002	0.255	0.453
SpO_2_ (%)	S (*n* = 40)	95.38 ± 2.04	98.74 ± 0.58^b^	99.82 ± 0.17	99.77 ± 0.21
	C (*n* = 40)	95.62 ± 3.22^a^	90.44 ± 2.74^a,b^	98.80 ± 0.48	99.51 ± 0.39
*t*		−0.411	21.024	0.532	0.065
*P*		0.682	7.17 × 10^−34^	0.496	0.961

Note: ^a^Indicates that the *T*0 moment is compared with each point in the group, and *P* < 0.05; ^b^indicates *P* < 0.05 compared with each time point between the groups.

**Figure 4:**
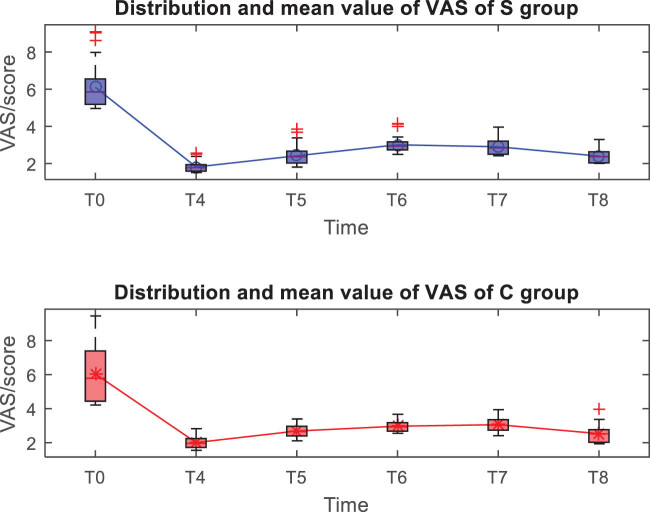
Distribution and mean value of VAS of the two groups.

The distribution and mean value of VAS scores of patients in the two groups after operation are shown in Figure 4. There is no significant difference in VAS scores between the two groups at each moment after surgery (*P* > 0.05) (Table 3).

**Table 3:** Comparison of VAS scores at different postoperative moments between the two groups ( \[\bar{x}\pm s]\] , points)

Index	Group	*T*0	*T*4	*T*5	*T*6	*T*7	*T*8
VAS	S (*n* = 40)	6.18 ± 1.22*	1.84 ± 0.32	2.32 ± 0.51	2.93 ± 0.44	2.87 ± 0.45	2.47 ± 0.46
	C (*n* = 40)	5.95 ± 1.74*	1.98 ± 0.43	2.54 ± 0.43	2.97 ± 0.42	2.91 ± 0.50	2.46 ± 0.53
*t*		0.599	−1.667	−1.736	−0.343	−0.417	0.066
*P*		0.551	0.099	0.086	0.732	0.678	0.947

*Indicates *P* < 0.05 compared to each time in the group.

There are significant differences in the number of vasopressor applications, length of hospital stay, and QoR-15 scores between the two groups (*P* < 0.05) (Table 4).

**Table 4:** Comparison of vasopressor doses, length of hospital stay, and QoR-15 scores between the two groups (*n* = 40, \[\bar{x}\pm s]\] )

Index	S group	C group	*t*	*P*
Number of vasopressor applications (times)	0.00 ± 0.00	0.70 ± 0.14	−35.343	9.23 × 10^−50^
Length of hospital stay (days)	10.26 ± 3.36	15.28 ± 2.54	−7.530	7.62 × 10^−11^
QoR-15 rating (points)	142.47 ± 5.83	127.64 ± 6.86	10.826	3.27 × 10^−16^

There is no significant difference in the incidence of intraoperative nausea and vomiting, hyperalgesia, and delirium between the two groups (*P* > 0.05). There are significant differences in the incidence of total adverse reactions and the incidence of hypotension (*P* < 0.05) (Table 5).

**Table 5:** Comparison of the incidence of adverse reactions between the two groups (*n* (%))

Index		S group	C group	*χ* ^2^	*P*
Occurrence of adverse reactions	Nausea and vomiting	1 (2.5)	2 (5.0)	0.346	0.556
	Hypotension	0 (0.0)	28 (70.0)	43.077	5.26 × 10^−11^
	Hyperalgesia	0 (0.0)	1 (2.5)	1.013	0.314
	Delirium	0 (0.0)	1 (2.5)	1.013	0.314
	Total incidence of adverse reactions	1 (2.5)	32 (80.0)	49.568	1.92 × 10^−12^

## 4 Discussion

Due to the advanced age, organ function, and compensatory function of elderly patients, often accompanied by cardiovascular and cerebrovascular diseases, they are more sensitive to anesthesia drugs, and are high-risk groups of anesthesia, and need to complete the operation under a safe and reliable anesthesia program. Nerve block has the advantages of small impact on circulatory, respiratory, and physiological functions, and has been widely reported in elderly hip surgery and lower limb surgery, especially for elderly patients with many comorbidities, but nerve block alone can easily lead to block insufficiency and cannot meet the needs of surgery [7]. Ultrasound-guided fascia iliac space block can effectively block the innervated femoral nerve and lateral femoral cutaneous nerve in the PFNA surgical area, and has the advantages of accurate positioning, fast onset, long duration of blockade, and hemodynamic stability [8]. Esketamine has sedative, analgesic, and anesthetic effects and has a positive effect on the patient’s postoperative mood [9]. Therefore, the combination of ultrasound-guided fascia iliac space block and esketamine in elderly PFNA should have obvious advantages.

Both anesthesia regimens were found to be effective, patients completed surgery as planned, and VAS scores indicated that postoperative analgesia was accurate in all patients. Comparing the vital signs at each moment, it was found that the HR, MAP, and SpO_2_ indicators in the experimental group were stable at each moment, the HR, MAP, and SpO_2_ in the control group at the *T*1 moment decreased significantly, and the *T*2 and *T*3 gradually recovered, which should be related to the control group’s use of opioids combined with propofol to maintain anesthesia. Literature [10,11] studies have shown that ultrasound-guided fascia iliac space block accurately and safely delivers anesthetics to the vicinity of the peripheral nerve trunk in the PFNA surgery area, which can effectively block the impulse conduction of nerves, and basically does not affect the patient’s intraoperative BP, HR and pulse, and other vital signs, while in this study, both groups of patients used fascial iliac space block, and the blocking method and medication were completely consistent; therefore, the impact of nerve block on the hemodynamics of the two groups of patients could be excluded. Refentanil is a powerful anesthetic analgesic drug, while propofol is a short-acting alkylphenol intravenous anesthetic drug, both have the advantages of fast onset, short maintenance time, rapid postoperative recovery, etc. Clinically, the two drugs are used in combination, but these two drugs have a certain inhibitory effect on the patient’s breathing, circulatory system, etc., and the degree of inhibition is obviously positively correlated with the dose of anesthetic drugs [12]. Thus, after the control group was given a loading dose of refentanil and propofol, the inhibitory effect of the two produced a synergistic effect, resulting in a transient decrease in BP and pulse oxygen. The above results also show that the experimental group can better maintain the hemodynamic stability of patients, because esketamine has sympathetic excitatory effect, which can neutralize the inhibitory effect caused by propofol drugs, stabilize circulation, control BP, and maintain stable vital signs [13,14].

Relevant studies have shown that opioids, while effectively exerting analgesic effects, inevitably bring adverse reactions such as nausea and vomiting, hypotension, hyperalgesia, and delirium due to dose dependence [15]. The results showed that the experimental group significantly reduced the incidence of adverse reactions, especially the absence of hypotension, hyperalgesia, and delirium, suggesting that it was safe and reliable. The clinical adverse reactions of esketamine are similar to ketamine, such as easily inducing mental reactions, and are dose related. Although the incidence rate of the clinical adverse reactions of esketamine is significantly lower than that of ketamine, it also limits the application of esketamine [16]. Two possible reasons are analyzed for the absence of hallucinations, delirium, and other psychological reactions in the experimental group. On the one hand, it is related to the combined application of propofol. Due to the activation of γ-aminobutyric acid receptors by propofol, the mental reactions such as hallucinations and delirium could be effectively suppressed [17]. On the other hand, the dose of esketamine used in this study is lower than the recommended dose of the drug (0.5 mg/kg). Literature [18] studies the effect of esketamine on the early postoperative cognitive function of elderly patients undergoing knee arthroplasty. The patient’s intravenous dose before skin incision is 0.5 mg/kg and the maintenance dose is 0.4 mg/kg/h, which is greater than that in our research. The conclusion in literature [18] is that no obvious adverse reactions are found.

Literature [19] shows that hypotension is an independent risk factor for postoperative delirium, but delirium did not occur in the experimental group, and the number of cases of hypotension in the intraoperative use of vasopressors and adverse reactions was significantly lower than that in the control group, which further verified that esketamine combined with fascia iliac block can maintain hemodynamic stability in patients. For the experimental group did not appear hyperalgesia and the control group had one patient, because esketamine can block the activity of spinal NMDA receptor and effectively inhibit central pain sensitization [20], remifentanil can enhance spinal NMDA receptor activity and induce central pain sensitization, especially continuous infusion during the anesthesia maintenance phase is a high-risk factor for refentanil-induced hyperalgesia [20,21]. The literature [22,23] also suggests that esketamine is effective in preventing hyperalgesia caused by refentanil. From the fact that the hospitalization time of patients in the experimental group was significantly less than that of the control group, and the QoR-15 score was significantly better than that of the control group, it can also be directly seen that the quality of recovery of patients in the experimental group is better, which is more conducive to the rapid recovery of patients.

In summary, fascial iliac block combined with esketamine intravenous general anesthesia in elderly patients can provide safe and reliable anesthesia effect for elderly patients, simple and easy operation, more stable intraoperative hemodynamics, and does not increase the incidence of adverse reactions, shortens the hospital stay, is conducive to the postoperative rehabilitation of elderly patients, and provides a safe and effective anesthesia method for elderly patients to undergo PFNA surgery.

## References

[1] Kulachote N, Sa-ngasoongsong P, Sirisreetreerux N, Chulsomlee K, Thamyongkit S, Wongsak S. Predicting factors for return to prefracture ambulatory level in high surgical risk elderly patients sustained intertrochanteric fracture and treated with proximal femoral nail antirotation (PFNA) with and without cement augmentation. Geriatr Orthop Surg Rehabil. 2020;11:21. 10.1177/2151459320912121 SAGE Publications Inc. PMC7068744 32201631

[2] Yan M, Kuang L, Ni J, Wang J, Huang J, Song D. Use of a double reverse traction repositor versus a traction table for the treatment of intertrochanteric femur fractures: a comparative study. Orthop Surg. 2021;13:1254–61. 10.1111/os.12956. PMC8274170 33951333

[3] Wu D, Ren G, Peng C, Zheng X, Mao F, Zhang Y. InterTan nail versus Gamma3 nail for intramedullary nailing of unstable trochanteric fractures. Diagn Pathol. 2014;9(1):1–6. 10.1186/s13000-014-0191-y BioMed Central Ltd. PMC4193997 25269555

[4] Zirngibl B, Biber R, Bail HJ. How to prevent cut-out and cut-through in biaxial proximal femoral nails: is there anything beyond lag screw positioning and tip-apex distance? Int Orthop. 2013;37(7):1363–8. 10.1007/s00264-013-1898-1. PMC3685663 23649496

[5] Sharma A, Mahajan A, John B. A comparison of the clinico-radiological outcomes with proximal femoral nail (PFN) and proximal femoral nail antirotation (PFNA) in fixation of unstable intertrochanteric fractures. J Clin Diagn Res. 2017;11(7):RC05–9. 10.7860/JCDR/2017/28492.10181. PMC5583812 28892987

[6] Stark P, Myles P, Burke J. Development and psychometric evaluation of a postoperative quality of recovery score the QoR-15. Anesthesiology. 2013;118(6):1332–40. 10.1097/ALN.0b013e318289b84b. 23411725

[7] Maurtua M, Fernando M, Finnegan P, Mehta B, Wu J, Foss J, et al. Use of the CTrach Laryngeal Mask Airway in adult patients: a retrospective review of 126 cases. J Clin Anesth. 2012;24:370–2. 10.1016/j.jclinane.2011.10.007. 22575604

[8] Vaughan B, Manley M, Stewart D, Iyer V. Distal injection site may explain lack of analgesia from fascia iliaca block for total hip. Reg Anesth Pain Med. 2013;38:556–7. 10.1097/AAP.0000000000000011. 24153047

[9] Pérez-Ruixo C, Rossenu S, Zannikos P, Nandy P, Singh J, Drevets W, et al. Population pharmacokinetics of esketamine nasal spray and its metabolite noresketamine in healthy subjects and patients with treatment-resistant depression. Clin Pharmacokinet. 2020;60(4):501–16. 10.1007/s40262-020-00953-4. 33128208

[10] Boghdady GW, Shalaby M. Safety and reliability of external fixation for basicervical and intertrochanteric fractures in high-risk elderly patients. Strateg Trauma Limb Reconstr. 2007;2(2–3):83–9. 10.1007/s11751-007-0025-5. PMC2322840 18427749

[11] Biddle C, Ford V. The neurotoxicity of general anesthetic drugs: emphasis on the extremes of age. Annu Rev Nurs Res. 2017;35:201–19. 10.1891/0739-6686.35.201. 27935781

[12] Schonberger R, Bardia A, Dai F, Michel G, Yanez D, Curtis J, et al. Variation in propofol induction doses administered to surgical patients over age 65. J Am Geriatr Soc. 2021;69(8):2195–209. 10.1111/jgs.17139. PMC8373684 33788251

[13] Javorcikova Z, Dangoisse M, Nikis S, Lechat JP, Gillain A, Fils JF, et al. The place of S-ketamine in fibromyalgia treatment (ESKEFIB): study protocol for a prospective, single-center, double-blind, randomized, parallel-group, dose-escalation controlled trial. Trials BioMed Cent Ltd. 2021;22(1):853. 10.1186/s13063-021-05814-4. PMC8627027 34838114

[14] Eberl S, Koers L, Van Hooft J, De Jong E, Hermanides J, Hollmann MW, et al. The effectiveness of a low-dose esketamine versus an alfentanil adjunct to propofol sedation during endoscopic retrograde cholangiopancreatography: a randomised controlled multicentre trial. Eur J Anaesthesiol. 2020;37(5):394–401. 10.1097/EJA.0000000000001134 Lippincott Williams and Wilkins. 31860599

[15] Oderda G, Gan T, Johnson B, Robinson S. Effect of opioid-related adverse events on outcomes in selected surgical patients. J Pain Palliat Care Pharmacother. 2013;27(1):62–70. 10.3109/15360288.2012.751956. 23302094

[16] Kaur U, Pathak BK, Singh A, Chakrabarti SS. Esketamine: a glimmer of hope in treatment-resistant depression. European Archives of Psychiatry and Clinical Neuroscience Springer Science and Business Media Deutschland GmbH. Vol. 271, 2021. p. 417–29. 10.1007/s00406-019-01084-z 31745646

[17] Lundy PM. Mechanism of action of ketamine. Br J Anaesth. 1980;52(6):638. 10.1093/bja/52.6.638 7426237

[18] Zhu X, Chen Q. Effect of esketamine on early postoperative cognitive function in elderly patients udergoning knee replacement. Jiangsu Med J. 2023;49(1):54–8.

[19] Maheshwari K, Ahuja S, Khanna A, Mao G, Perez-Protto S, Farag E, et al. Association between perioperative hypotension and delirium in postoperative critically ill patients: a retrospective cohort analysis. Anesth Analg. 2019;130:1. 10.1213/ANE.0000000000004517. 31725024

[20] Ma P, Chen P, Zhou ZL, Mo RF, Wu M, Song XJ. Activation of EphB receptors contributes to primary sensory neuron excitability by facilitating Ca2+ influx directly or through Src kinase-mediated N-methyl-d-aspartate receptor phosphorylation. Pain. 2020;161(7):1584–96. 10.1097/j.pain.0000000000001855 Lippincott Williams and Wilkins. 32149862

[21] Koo C-H, Yoon S, Kim B-R, Cho Y, Kim TK, Jeon Y, et al. Intraoperative naloxone reduces remifentanil-induced postoperative hyperalgesia but not pain: a randomized controlled trial. Br J Anaesth. 2017;119(6):1161–8. 10.1093/bja/aex253. 29029049

[22] Koppert W, Sittl R, Scheuber K, Alsheimer M, Schmelz M, Schüttler J. Differential modulation of remifentanil-induced analgesia and postinfusion hyperalgesia by S-ketamine and clonidine in humans [En ligne]; 2003 [cité le. Disponible. http://pubs.asahq.org/anesthesiology/article-pdf/99/1/152/408263/0000542-200307000-00025.pdf. 10.1097/00000542-200307000-00025 12826855

[23] Li S, Zeng J, Wan X, Yao Y, Wu Y, Zhao N, et al. Enhancement of spinal dorsal horn neuron N-methyl-d-aspartate receptor phosphorylation as the mechanism of remifentanil-induced hyperalgesia: roles of protein kinase C and calcium/calmodulin-dependent protein kinase II. Mol Pain. 2017;13:1744806917723789. 10.1177/1744806917723789 SAGE Publications Inc. PMC5549877 28714352

